# KT-SRAF-LVD-Based Signal Coherent Integration Method for High-Speed Target Detecting in Airborne Radar

**DOI:** 10.3390/s25072128

**Published:** 2025-03-27

**Authors:** Wenwen Xu, Yuhang Wang, Jianyin Cao, Hao Wang

**Affiliations:** School of Electronic and Optical Engineering, Nanjing University of Science and Technology, Nanjing 210094, China; wenwenxu@njust.edu.cn (W.X.); wyh15857@gmail.com (Y.W.); jianyin.cao@njust.edu.cn (J.C.)

**Keywords:** high-speed target, motion error, range migration, Doppler frequency migration, long-term coherent integration

## Abstract

In the application of an airborne radar platform, the rapid relative motion between target and airborne radar induces range migration (RM) and Doppler frequency migration (DFM). The motion errors caused by airflow, air friction, and navigation inaccuracies will exacerbate the RM and DFM problems and render traditional coherent integration methods ineffective. Previously reported airborne coherent integration methods are hindered by high computational complexity, limiting their practical application. Therefore, developing motion error compensation and coherent integration methods with reduced computational complexity and a high detection performance is of critical importance. To address these challenges, a novel method based on the keystone transform, sequence-reversing autocorrelation function, and Lv’s distribution (KT-SRAF-LVD) is proposed. Simulation results demonstrate that the proposed method achieves a good balance between computational complexity and detection performance, indicating great potential for practical engineering applications.

## 1. Introduction

In radar target detection, long-term coherent integration (LTCI) techniques are widely employed to enhance the signal-to-noise ratio (SNR) and improve the detection capability for weak targets [1,2,3]. However, during the coherent processing interval (CPI), the motion of high-speed targets can induce both range migration (RM) and Doppler frequency migration (DFM), resulting in significant performance degradation in coherent integration [4,5,6,7]. Compared to ground-based radar, airborne radar overcomes the limitations imposed by Earth’s curvature, extending detection capabilities for low and ultra-low altitudes. It is widely employed in applications such as space target detection, long-range early warning, and reconnaissance, playing an irreplaceable role in the detection of high-speed targets [8]. During application, the external disturbances during flight will cause significant motion errors of aircraft deviating from ideal straight-line motion [9,10]. These motion errors directly impact the phase and amplitude of the echo signal, further exacerbating RM and DFM. As a result, additional losses will increase during coherent integration.

Substantial research efforts have been devoted to long-term coherent integration techniques for high-speed target signals. For uniformly moving targets, common methods for RM correction include the keystone transform (KT) [11], Radon–Fourier transform (RFT) [12], scaled inverse Fourier transform (SCIFT) [13], sequence-reversing transform (SRT) [14], etc. For targets undergoing uniform acceleration, the generalized RFT (GRFT) is one of the most representative methods [15]. The GRFT method simultaneously searches for the motion parameters of targets while demonstrating excellent noise immunity. However, its three-dimensional exhaustive search mechanism results in a high computational complexity, limiting its practical applicability. Another approach is to first correct for RM and then model the target echo signal as a linear frequency-modulated (LFM) signal in the slow time domain. The target acceleration is estimated by determining the chirp rate of the LFM signal. Several notable methods have been developed for this purpose, including the cubic phase function (CPF) [16,17], parameterized centroid frequency–chirp rate distribution (PCFCRD) [18], second-order Wigner–Ville distribution (SWVD) [19], Lv’s distribution (LVD) [20], and the symmetric autocorrelation function-scaled Fourier transform (SAF-SFT) [21], etc. Another approach is to estimate the motion parameters of targets simultaneously, such as with the three-dimensional scaled transform (TDST) [22] and three-dimensional coherent integration (TDCI) method [23]. These works only considered fixed radar platforms and did not consider the motion errors caused by the motion of the airborne platform.

Motion error compensation for airborne radar primarily includes methods based on aerial attitude and echo data. Aerial attitude-based compensation [24,25] relies on motion parameters recorded by devices such as the inertial measurement unit (IMU) and global positioning system (GPS). In addition, decoupling techniques are applied for the point-by-point compensation of two-dimensional space-variant errors. However, its performance is influenced by the accuracy of the devices and environmental interference. Echo-based compensation [26,27,28] estimates errors by analyzing echo characteristics, with techniques such as image contrast optimization and waveform entropy minimization. However, under low signal-to-noise ratio (SNR) conditions, the difficulty in extracting echo characteristics can significantly degrade compensation accuracy and imaging quality.

The improved GRFT (IGRFT) method was proposed to address the impact of airborne platform motion errors on the coherent integration of echo signals from high-speed targets [29]. By extending the GRFT by incorporating a platform motion error search, the IGRFT method achieves coherent integration with excellent detection performance. However, the five-dimensional search mechanism results in a high computational complexity, making it impractical for applications.

This paper proposes a sequence-reversing autocorrelation function (SRAF) to estimate sinusoidal motion error parameters and combines it with the traditional KT-LVD method [30] to obtain the KT-SRAF-LVD method. First, the RM is corrected using the KT. Next, the parameters of the sinusoidal motion error are jointly estimated after performing an SRAF operation, thereby enabling the correction of the airborne platform motion error. Finally, the LVD method is applied to estimate the equivalent acceleration and construct a phase compensation function for DFM correction. Detailed experiments are provided to demonstrate the effectiveness of the proposed method. The results show that the proposed method achieves a good balance between computational complexity and detection performance.

The rest of this paper is organized as follows. In Section 2, a signal model is established. The proposed method is described in detail in Section 3. Section 4 provides an analysis of the cross terms and computational complexity. The simulation results are discussed in Section 5. Finally, conclusions are presented in Section 6.

## 2. Signal Model

The narrowband LFM signal emitted by the radar can be expressed as

(1) $$s_{t}(t)=A_{t}\mathrm{rect}\left(\frac{t}{T_{p}}\right)\exp\left(j2\pi f_{c}t+j\pi \mu t^{2}\right),$$

where $\mathrm{rect}\left(\frac{t}{T_{p}}\right)=\left\{\begin{array}{c} 1,\left|t\right|\leq T_{p}/2\\ 0,\left|t\right|>T_{p}/2 \end{array}\right.$, t is the fast time, $f_{c}$ is the carrier frequency, $T_{P}$ is the pulse width, μ is the frequency-modulated rate, and $A_{t}$ is the amplitude of the transmitted signal.

After down-conversion, the echo signal can be expressed as

(2) $$s_{r}\left(t,t_{m}\right)=A_{r}\mathrm{rect}\left\{\frac{1}{T_{P}}\left[t-\frac{2R(t_{m})}{c}\right]\right\}\exp\left[-j\frac{4\pi R(t_{m})}{\lambda }\right]\exp\left\{j\pi \mu {\left[t-\frac{2R(t_{m})}{c}\right]}^{2}\right\},$$

where $A_{r}$ is the amplitude of the received signal, c is the light speed, $t_{m}=m\cdot T_{r}$ is the slow time, $T_{r}$ is the pulse repetition time (PRT), m=−M/2,…,M/2 is the transmitted pulse number index, and M is the number of coherent integrated pulses and is assumed to be even. $R(t_{m})$ is the radial distance between the target and the airborne radar, and $\lambda =c/f_{c}$ is the signal wavelength.

**Figure 1:**
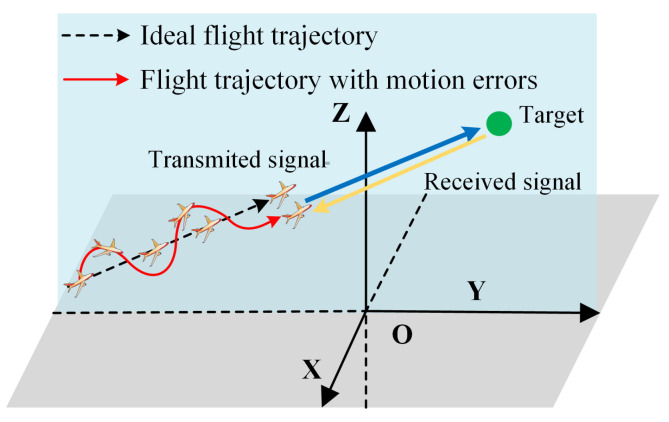
Geometrical schematic of a radar system with motion errors.

In practice, the external disturbances and uncertainty of platform movement during flight will cause significant motion errors, leading the aircraft to deviate from its ideal straight-line motion, as shown in Figure 1 [29].

Three classical models commonly used to represent motion errors include linear error, quadratic error, and sinusoidal error [31,32]. The linear and quadratic errors correspond to changes in the relative velocity and acceleration between the airborne radar and the target, which do not affect the corrections of RM and DFM in existing LTCI methods. Therefore, for a target in uniformly accelerated motion, the radial distance between the target and the radar can be expressed as

(3) $$R(t_{m})=R_{0}+v_{0}t_{m}+\frac{1}{2}a{t_{m}}^{2}+A_{e}\sin\;\left(2\pi f_{e}t_{m}\right),$$

where $R_{0}$ denotes the initial slant range, $v_{0}$ denotes the equivalent velocity when a linear error exists, and a denotes the equivalent acceleration when a quadratic error exists. $A_{e}$ and $f_{e}$ represent the amplitude and frequency of sinusoidal error, respectively.

After pulse compression (PC), Equation (2) can be expressed as

(4) $$s_{p}\left(t,t_{m}\right)=A_{0}\sin c\langle B\left\{t-\frac{2\left[R_{0}+v_{0}t_{m}+\frac{1}{2}a{t_{m}}^{2}+A_{e}\sin\left(2\pi f_{e}t_{m}\right)\right]}{c}\right\}\rangle \exp\left\{-j\frac{4\pi }{\lambda }\left[R_{0}+v_{0}t_{m}+\frac{1}{2}a{t_{m}}^{2}+A_{e}\sin\left(2\pi f_{e}t_{m}\right)\right]\right\},$$

where $A_{0}$ is the amplitude of the pulse-compressed signal, and B is the signal bandwidth.

Equation (4) indicates that the target’s envelope and Doppler frequency gradually vary with slow time, potentially leading to RM and DFM. When the RM and DFM exceed a single resolution cell, they disrupt coherent integration, leading to a decline in overall performance.

The following presents an analysis of sinusoidal error. The radial range and Doppler frequency affected by the sinusoidal error are given by

(5) $$R_{s}\left(t_{m}\right)=A_{e}\sin\left(2\pi f_{e}t_{m}\right).$$

(6) $$f_{\mathrm{ds}}(t_{m})=\frac{1}{2\pi }\cdot \frac{d\left\{-\frac{4\pi }{\lambda }\left[A_{e}\sin\left(2\pi f_{e}t_{m}\right)\right]\right\}}{dt_{m}}=-\frac{2}{\lambda }\left[2\pi A_{e}f_{e}\cos\left(2\pi f_{e}t_{m}\right)\right].$$

The coherent integration is impacted by sinusoidal error when the following condition is satisfied

(7) $$\left|\max\left[R_{s}(t_{m})\right]-\min\left[R_{s}(t_{m})\right]\right|\approx 2\left|A_{e}\right|>\Delta R,$$

(8) $$\left|\max\left[f_{\mathrm{ds}}(t_{m})\right]-\min\left[f_{\mathrm{ds}}(t_{m})\right]\right|\approx \frac{8\pi }{\lambda }\left|A_{e}f_{e}\right|>\Delta f_{d},$$

where ΔR represents the size of the range cell, and $\Delta f_{d}$ represents the size of the Doppler frequency cell.

In practice, $A_{e}$ is within the range of [−1, 1], typically less than a range cell, indicating that the impact of sinusoidal error on RM can be neglected. However, $8\pi \left|A_{e}f_{e}\right|/\lambda$ can be much larger than a Doppler-frequency resolution cell, meaning that its impact on coherent integration cannot be ignored. To achieve the coherent integration of multi-pulse echo signals for high-speed maneuvering targets, it is essential to correct the sinusoidal error.

The impact of sinusoidal error on RM and DFM is simulated below. The radar system parameters, equivalent motion parameters, and sinusoidal error parameters are presented in Table 1, Table 2 and Table 3, respectively.

**Figure 2:**
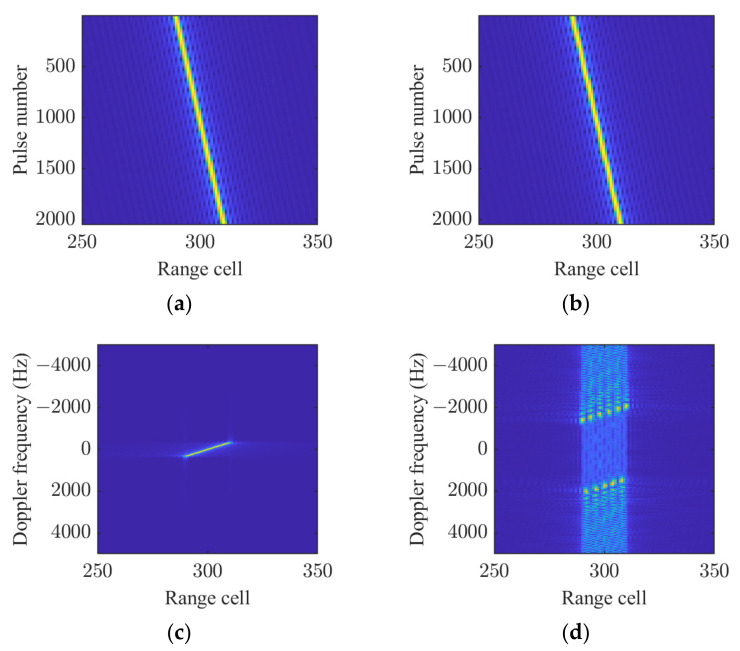
Impact of sinusoidal motion error. (**a**) RM without sinusoidal error. (**b**) RM with sinusoidal error. (**c**) Coherent integration result without sinusoidal error. (**d**) Coherent integration result with sinusoidal error.

The results after PC and coherent integration, both with and without sinusoidal error, are shown in Figure 2. It can be observed that the acceleration causes significant DFM, and the sinusoidal error severely exacerbates DFM, preventing the signal energy from being properly concentrated. However, the sinusoidal error does not have a significant impact on RM.

## 3. Principle of KT-SRAF-LVD

### 3.1. Principle of KT

After performing the fast Fourier transform (FFT) on Equation (4) with respect to t, the result can be expressed as

(9) $$s_{p}\left(f,t_{m}\right)=A\mathrm{rect}\left(\frac{f}{B}\right)\exp\left\{-j\frac{4\pi \left(f+f_{c}\right)}{c}\left[R_{0}+v_{0}t_{m}+\frac{1}{2}a{t_{m}}^{2}+A_{e}\sin\left(2\pi f_{e}t_{m}\right)\right]\right\},$$

where f denotes the range frequency, and A denotes the amplitude after FFT with respect to t.

The existence of a linear and quadratic coupling between f and $t_{m}$ in Equation (9) may affect the coherent integration. Therefore, the KT is performed on Equation (9) to eliminate the linear coupling, whose scaling formula is expressed as

(10) $$t_{m}=\frac{f_{c}}{f+f_{c}}t_{m}^{\prime },$$

where $t_{m}^{\prime }$ is the scaled slow time.

Applying the KT to Equation (9) yields

(11) $$s_{p}\left(f,t_{m}^{\prime }\right)=A\mathrm{rect}\left(\frac{f}{B}\right)\exp\langle -j\frac{4\pi \left(f+f_{c}\right)}{c}\left\{R_{0}+A_{e}\sin\left[2\pi f_{e}\left(\frac{f_{c}}{f+f_{c}}\right)t_{m}^{\prime }\right]\right\}\rangle \exp\left(-j\frac{4\pi f_{c}}{c}v_{0}t_{m}^{\prime }\right)\exp\left[-j\frac{2\pi }{c}\left(\frac{{f_{c}}^{2}}{f+f_{c}}\right)a{t_{m}^{\prime }}^{2}\right].$$

Under the narrow band environment $f\ll f_{c}$, we have $f_{c}/\left(f_{c}+f\right)\approx 1$ [30]. Therefore, Equation (11) can be rewritten as

(12) $$s_{p}\left(f,t_{m}^{\prime }\right)=A\mathrm{rect}\left(\frac{f}{B}\right)\exp\left\{-j\frac{4\pi \left(f+f_{c}\right)}{c}\left[R_{0}+A_{e}\sin\left(2\pi f_{e}t_{m}^{\prime }\right)\right]\right\}\exp\left(-j\frac{4\pi f_{c}}{c}v_{0}t_{m}^{\prime }\right)\exp\left(-j\frac{2\pi f_{c}}{c}a{t_{m}^{\prime }}^{2}\right).$$

The coupling between range frequency f and slow time $t_{m}$ is removed, which effectively corrects RM without requiring prior knowledge of the target’s velocity. When the Doppler frequency corresponding to the target equivalent velocity exceeds half of the pulse repetition frequency (PRF), undersampling occurs [30]. In this case, the velocity can be expressed as

(13) $$v_{0}=v_{u}+nv_{m},$$

where n is the fold factor, $f_{p}$ is the PRF, $v_{m}=\lambda f_{p}/2$ is the blind velocity, and $v_{u}=\mathrm{mod}(v_{0},v_{m})$ is the unambiguous velocity that satisfies $v_{u}<v_{m}/2$.

To compensate for undersampling, it is necessary to search for the folding factor and construct a phase compensation function for undersampling compensation, thereby mitigating the residual RM in the echo signal. The principle of undersampling compensation has already been provided in [30] and will not be elaborated here.

In Equation (12), $\exp[-j4\pi A_{e}f\sin(2\pi f_{e}t_{m}^{\prime })/c]$ represents the RM caused by sinusoidal error, which was previously mentioned as negligible. So Equation (12) can be rewritten as

(14) $$s_{p}\left(f,t_{m}^{\prime }\right)=A\mathrm{rect}\left(\frac{f}{B}\right)\exp\left(-j\frac{4\pi f}{c}R_{0}\right)\exp\left\{-j\frac{4\pi }{\lambda }\left[R_{0}+v_{0}t_{m}^{\prime }+\frac{1}{2}a{t_{m}^{\prime }}^{2}+A_{e}\sin\left(2\pi f_{e}t_{m}^{\prime }\right)\right]\right\}.$$

After applying the inverse FFT (IFFT) on Equation (14) with respect to f, the result is given by

(15) $$s_{p}\left(t,t_{m}^{\prime }\right)=A_{0}\sin c\left[B\left(t-\frac{2R_{0}}{c}\right)\right]\exp\left\{-j\frac{4\pi }{\lambda }\left[R_{0}+v_{0}t_{m}^{\prime }+\frac{1}{2}a{t_{m}^{\prime }}^{2}+A_{e}\sin\left(2\pi f_{e}t_{m}^{\prime }\right)\right]\right\}.$$

As observed from Equation (15), the signal energy is now concentrated within a single range cell. To ensure that the echo energy after coherent integration is also concentrated within a single Doppler cell, further compensation for the DFM caused by acceleration and sinusoidal error is necessary.

### 3.2. Principle of Sinusoidal Error Compensation

After RM correction, a preliminary screening of range cells where targets may exist is performed using non-coherent integration, which can be expressed as

(16) $$\hat{R_{0}}=\mathrm{round}\left\{\frac{c}{2\Delta R}\cdot \underset{t}{\mathrm{argmax}}\left[\sum_{t_{m}^{\prime }}\left|s_{p}\left(t,t_{m}^{\prime }\right)\right|\right]\right\},$$

where $\hat{R_{0}}$ is the estimated range cell index.

The extract signal along the range cell can be expressed as

(17) $$s_{\mathrm{ext}}\left(t_{m}^{\prime }\right)=A_{1}\exp\left\{-j\frac{4\pi }{\lambda }\left[v_{0}t_{m}^{\prime }+\frac{1}{2}a{t_{m}^{\prime }}^{2}+A_{e}\sin\left(2\pi f_{e}t_{m}^{\prime }\right)\right]\right\},$$

where $A_{1}=A_{0}\exp\left(-j4\pi R_{0}/\lambda \right)$.

If the LVD is directly used to estimate the acceleration at this time, the phase factor $\exp\left[-j4\pi A_{e}\sin\left(2\pi f_{e}t_{m}^{\prime }\right)/\lambda \right]$ will cause the spectrum of the result to broaden significantly, preventing energy from being concentrated. To solve this problem, this paper proposes an SRAF, which can be expressed as

(18) $$s_{\mathrm{SRAF}}\left(t_{m}^{\prime }\right)=s_{\mathrm{ext}}\left(t_{m}^{\prime }\right)s_{\mathrm{ext}}^{*}\left(-t_{m}^{\prime }\right).$$

Substituting Equation (17) into Equation (18), the result is given by

(19) $$s_{\mathrm{SRAF}}\left(t_{m}^{\prime }\right)={A_{1}}^{2}\exp\left\{-j\frac{8\pi }{\lambda }\left[v_{0}t_{m}^{\prime }+A_{e}\sin\left(2\pi f_{e}t_{m}^{\prime }\right)\right]\right\}.$$

A phase compensation function is constructed and multiplied with Equation (19), which can be expressed as

(20) $$H_{1}(A_{\mathrm{test}},f_{\mathrm{test}})=\exp\left[j\frac{8\pi }{\lambda }A_{\mathrm{test}}\sin\left(2\pi f_{\mathrm{test}}t_{m}^{\prime }\right)\right].$$

Then, the result is given by

(21) $$s_{\mathrm{SRAF}}^{\prime }\left(t_{m}^{\prime };A_{\mathrm{test}},f_{\mathrm{test}}\right)={A_{1}}^{2}\exp\left(-j\frac{8\pi }{\lambda }v_{0}t_{m}^{\prime }\right)\exp\left\{-j\frac{8\pi }{\lambda }\left[A_{e}\sin\left(2\pi f_{e}t_{m}^{\prime }\right)-A_{\mathrm{test}}\sin\left(2\pi f_{\mathrm{test}}t_{m}^{\prime }\right)\right]\right\}.$$

Performing the FFT on Equation (21) with respect to $t_{m}^{\prime }$, the result of $\exp(-j8\pi v_{0}t_{m}^{\prime }/\lambda )$ is given by $\delta (f_{t_{m}^{\prime }}+4v_{0}/\lambda )$, where $f_{t_{m}^{\prime }}$ is the frequency with respect to $t_{m}^{\prime }$. However, the sinusoidal error prevents the concentration of energy. When $A_{\mathrm{test}}=A_{e}$, $f_{\mathrm{test}}=f_{e}$, only then can the signal energy be concentrated. Therefore, the estimated sinusoidal error parameters can thus be expressed as

(22) $$\left(\hat{A_{e}},\hat{f_{e}}\right)=\underset{\left(A_{\mathrm{test}},f_{\mathrm{test}}\right)}{\mathrm{argmax}}\left\{\max\left|\mathrm{FFT}\left[s_{\mathrm{SRAF}}^{\prime }\left(t_{m}^{\prime };A_{\mathrm{test}},f_{\mathrm{test}}\right)\right]\right|\right\}.$$

Based on the estimated parameters, a phase compensation function $\exp[j4\pi \hat{A_{e}}\sin(2\pi \hat{f_{e}}t_{m}^{\prime })/\lambda ]$ is constructed to correct for the sinusoidal error in Equation (15). When $\hat{A_{e}}=A_{e},\hat{f_{e}}=f_{e}$, the result is given by

(23) $$\;s_{p}^{\prime \prime }\left(t,t_{m}^{\prime }\right)=A_{0}\sin c\left[B\left(t-\frac{2R_{0}}{c}\right)\right]\exp\left[-j\frac{4\pi }{\lambda }\left(R_{0}+v_{0}t_{m}^{\prime }+\frac{1}{2}a{t_{m}^{\prime }}^{2}\right)\right].$$

### 3.3. Principle of LVD

After applying the sinusoidal error correction, Equation (17) is given by

(24) $$s_{\mathrm{ext}}^{\prime }\left(t_{m}^{\prime }\right)=A_{1}\exp\left[-j\frac{4\pi }{\lambda }\left(v_{0}t_{m}^{\prime }+\frac{1}{2}a{t_{m}^{\prime }}^{2}\right)\right].$$

The principle of the LVD can be expressed as

(25) $$s_{\mathrm{LVD}}\left(f_{t_{m}^{\prime }},f_{\tau }\right)={\mathrm{FFT}}_{\tau }\left\{\int s_{\mathrm{PSIAF}}\left(t_{m}^{\prime },\tau \right)\exp\left[-j2\pi f_{t_{m}^{\prime }}\frac{\left(\tau +\tau_{c}\right)t_{m}^{\prime }}{h}\right]dt_{m}^{\prime }\right\},$$

where τ is a lag-time variable with respect to $t_{m}^{\prime }$, $f_{\tau }$ is the frequency with respect to τ, and h is a scaling factor, typically set to 1 [20]. $s_{\mathrm{PSIAF}}\left(t_{m}^{\prime },\tau \right)$ is a parametric symmetric instantaneous autocorrelation function (PSIAF), which can be expressed as

(26) $$s_{\mathrm{PSIAF}}\left(t_{m}^{\prime },\tau \right)=s_{\mathrm{ext}}^{\prime }\left(t_{m}^{\prime }+\frac{\tau +\tau_{c}}{2}\right){s_{\mathrm{ext}}^{\prime }}^{*}\left(t_{m}^{\prime }-\frac{\tau +\tau_{c}}{2}\right).$$

Substituting Equation (24) into Equation (25), the result is given by

(27) $$s_{\mathrm{LVD}}\left(f_{t_{m}^{\prime }},f_{\tau }\right)=A_{2}\delta \left(f_{t_{m}^{\prime }}+\frac{2ah}{\lambda }\right)\delta \left(f_{\tau }+\frac{2v_{0}}{\lambda }\right),$$

where $A_{2}$ denotes the signal amplitude after LVD operation.

The result contains a unique peak at $\left(-2ah/\lambda ,-2v_{0}/\lambda \right)$, corresponding to the equivalent acceleration $\hat{a}$, which can be estimated through peak detection. Based on the estimated parameter, a phase compensation function $\exp(j2\pi \hat{a}{t_{m}^{\prime }}^{2}/\lambda )$ is constructed to correct for the DFM caused by acceleration in Equation (23). When $\hat{a}=a$, the result is given by

(28) $$\;s_{p}^{\prime \prime \prime }\left(t,t_{m}^{\prime }\right)=A_{0}\sin c\left[B\left(t-\frac{2R_{0}}{c}\right)\right]\exp\left[-j\frac{4\pi }{\lambda }\left(R_{0}+v_{0}t_{m}^{\prime }\right)\right].$$

The RM and DFM caused by the equivalent velocity and acceleration, as well as sinusoidal error of the airborne platform, are fully corrected. Coherent integration can be achieved by performing an FFT on Equation (28) with respect to $t_{m}^{\prime }$, which can be expressed as

(29) $$\;s_{p}^{\prime \prime \prime }\left(t,f_{t_{m}^{\prime }}\right)=A_{3}\sin c\left[B\left(t-\frac{2R_{0}}{c}\right)\right]\delta \left(f_{t_{m}^{\prime }}+\frac{2v_{0}}{\lambda }\right),$$

where $A_{3}=A_{0}\exp(-j4\pi R_{0}/\lambda )$ is a constant.

From Equation (29), it is observed that the result contains a unique peak at $\left(2R_{0}/c,-2v_{0}/\lambda \right)$. Finally, the constant false alarm rate (CFAR) detector is applied for target detection.

### 3.4. Detailed Procedure of the Proposed Method

**Figure 3:**
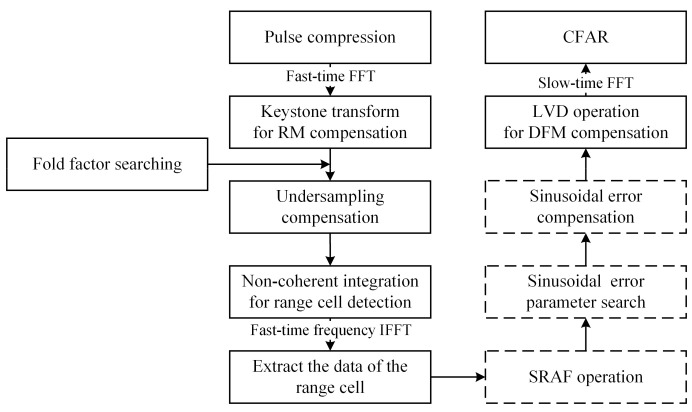
Flowchart of the proposed method.

A flowchart of the proposed KT-SRAF-LVD method is illustrated in Figure 3, where the dashed box indicates the improvements of this method over the KT-LVD method. The main implementation steps of the method are as follows:

- Step 1: Perform the FFT on the pulse-compressed signal $s_{p}(t,t_{m})$ with respect to t, yielding $s_{p}(f,t_{m})$;
- Step 2: Apply the KT to $s_{p}(f,t_{m})$ to correct the RM caused by unambiguous velocity, yielding $s_{p}(f,t_{m}^{\prime })$;
- Step 3: Perform a fold factor search on $s_{p}(f,t_{m}^{\prime })$ to correct the RM caused by undersampling, yielding $s_{p}^{\prime }(f,t_{m}^{\prime })$;
- Step 4: Apply IFFT on $s_{p}^{\prime }(f,t_{m}^{\prime })$ with respect to f, yielding $s_{p}^{\prime }(t,t_{m}^{\prime })$;
- Step 5: Detect the target’s range cell to extract the corresponding slow time dimension signal $s_{\mathrm{ext}}(t_{m}^{\prime })$;
- Step 6: Apply the SRAF operation to $s_{\mathrm{ext}}(t_{m}^{\prime })$ to obtain $s_{\mathrm{SRAF}}(t_{m}^{\prime })$. Then, search for the sinusoidal parameters $\hat{A_{e}}$ and $\hat{f_{e}}$;
- Step 7: Construct the phase compensation function $\exp[j4\pi \hat{A_{e}}\sin(2\pi \hat{f_{e}}t_{m})/\lambda ]$ to correct the DFM caused by sinusoidal error for $s_{p}^{\prime }(t,t_{m}^{\prime })$, yielding $s_{p}^{\prime \prime }(t,t_{m}^{\prime })$. At this stage, $s_{\mathrm{ext}}(t_{m}^{\prime })$ is transformed into $s_{\mathrm{ext}}^{\prime }(t_{m}^{\prime })$;
- Step 8: Apply the LVD operation to $s_{\mathrm{ext}}^{\prime }(t_{m}^{\prime })$ to estimate the target’s equivalent acceleration $\hat{a}$;
- Step 9: Construct the phase compensation function $\exp(j2\pi \hat{a}{t_{m}^{\prime }}^{2}/\lambda )$ to correct the DFM caused by acceleration for $s_{p}^{\prime \prime }(t,t_{m}^{\prime })$, yielding $s_{p}^{\prime \prime \prime }(t,t_{m}^{\prime })$;
- Step 10: Perform the FFT on $s_{p}^{\prime \prime \prime }(t,t_{m}^{\prime })$ with respect to $t_{m}^{\prime }$ to achieve coherent integration;
- Step 11: Apply the CFAR detector for target detection.

## 4. Performance Analysist

### 4.1. Cross Terms

For multiple targets, cross terms may arise between the echo signals of different targets, potentially affecting the performance of the method. The following analyzes the cross terms in the KT-SRAF-LVD method.

Since KT is a linear transformation, no cross terms will appear when processing multi-target signals. The analysis of cross terms in the LVD method has been comprehensively detailed in [20] and thus will not be further elaborated on here. Considering that the SRAF operation is introduced in this paper and its results are utilized for sinusoidal error compensation, the following section will analyze the cross terms in the SRAF operation.

When multiple targets are present within the same range cell, Equation (17) can be rewritten as

(30) $$s_{\mathrm{ext}}\left(t_{m}^{\prime }\right)=\sum_{i=1}^{k}A_{i1}\exp\left\{-j\frac{4\pi }{\lambda }\left[v_{0i}t_{m}^{\prime }+\frac{1}{2}a_{i}{t_{m}^{\prime }}^{2}+A_{e}\sin\left(2\pi f_{e}t_{m}^{\prime }\right)\right]\right\},$$

where $A_{i1}=A_{i}\exp\left(-j4\pi R_{0}/\lambda \right)$, $A_{i}$ denotes the signal amplitude of the i-th target, and a total of k targets are considered.

Applying the SRAF to Equation (30), the result can be expressed as

(31) $$s_{\mathrm{SRAF}}\left(t_{m}^{\prime }\right)=\sum_{i=1}^{k}{A_{i1}}^{2}\exp\left\{-j\frac{8\pi }{\lambda }\left[v_{0i}t_{m}^{\prime }+A_{e}\sin\left(2\pi f_{e}t_{m}^{\prime }\right)\right]\right\}+R_{\mathrm{cross}}\left(t_{m}^{\prime }\right),$$

where $R_{\mathrm{cross}}\left(t_{m}^{\prime }\right)$ represents the cross terms, which can be expressed as

(32) $$R_{\mathrm{cross}}\left(t_{m}^{\prime }\right)=\sum_{p=1}^{k}\sum_{q=1,q\neq p}^{k}A_{p1}A_{q1}\exp\left[-j\frac{4\pi }{\lambda }\left(v_{0p}+v_{0q}\right)t_{m}^{\prime }\right]\exp\left[-j\frac{4\pi }{\lambda }\left(a_{0p}-a_{0q}\right){t_{m}^{\prime }}^{2}\right]\exp\left[-j\frac{8\pi }{\lambda }A_{e}\sin\left(2\pi f_{e}t_{m}^{\prime }\right)\right].$$

In Equation (32), since $\exp[-j4\pi (a_{0p}-a_{0q}){t_{m}^{\prime }}^{2}/\lambda ]$ will cause additional DFM, during the search process of sinusoidal error parameters the cross term is suppressed and the energy cannot be completely concentrated. Thus, the SRAF has an effective inhibitory effect on the cross terms generated between different targets.

### 4.2. Computational Complexity

The SAF-SFT [20], IGRFT [29], and KT-LVD [30] methods are selected as comparison methods in this paper. The computational complexity of their main steps is analyzed based on the number of complex multiplications. The number of the pulse number, range cells, search fold factor number, search velocities, search accelerations, search sinusoidal error amplitudes, and search sinusoidal error frequencies are denoted by M, N, $N_{n}$, $N_{v}$, $N_{a}$, $N_{A}$ and $N_{f}$, respectively.

The SAF-SFT method involves two SAF-SFT operations; both can be rapidly implemented based on the chirp Z-transform (CZT), with a computational complexity of $O(M^{3}+M^{2}+3MN\log_{2}M+3M^{2}\log_{2}M+MN\log_{2}N+M^{2}\log_{2}M)$. The IGRFT method performs a five-dimensional joint search to obtain the equivalent motion parameters and the sinusoidal error parameters, resulting in a computational complexity of $O(N_{v}N_{a}N_{A}N_{f}MN)$. For the proposed KT-SRAF-LVD method, the computational complexity of the KT method based on CZT is $O(4MN\log_{2}M)$. Next, the computational complexity of the fold factor search is $O(N_{n}MN\log_{2}N)$. Then, the computational complexity of the SRAF and sinusoidal error parameter search is $O(N_{A}N_{f}M\log_{2}M)$. Finally, the computational complexity of the CZT-based LVD is $O(M^{2}+3MN\log_{2}M+MN\log_{2}N)$.

**Figure 4:**
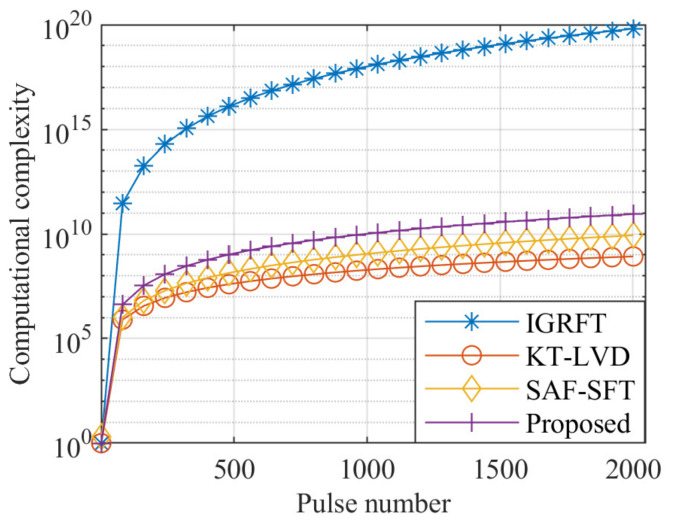
Computational complexity of each method.

Assuming $N_{v}=N_{a}=N_{A}=N_{f}=N=M$, the computational complexities of each method are presented in Table 4. Under the radar simulation parameters of Table 1 and Table 2, the blind velocity is 150 m/s, and the search range of the fold factor is [−5, 5], with $N_{n}=11$. The computational complexity of each method for varying pulse numbers is shown in Figure 4, where the pulse number ranges from 1 to 2048.

As shown in Figure 4, the IGRFT method involves multi-dimensional searches, resulting in a high computational complexity. In contrast, the proposed KT-SRAF-LVD method reduces the search dimensions by focusing solely on the fold factor and sinusoidal error parameters. This approach significantly lowers the computational complexity compared to the IGRFT method. However, the computational complexity of the proposed method is slightly higher than that of the SAF-SFT and KT-LVD methods.

## 5. Simulation Results

### 5.1. Coherent Integration for a Single Target

To validate the effectiveness of the proposed method, echo signals affected by airborne platform motion errors were simulated and analyzed using the method. The radar system parameters, equivalent motion parameters, and sinusoidal error parameters are consistent with those listed in Table 1, Table 2 and Table 3, respectively.

**Figure 5:**
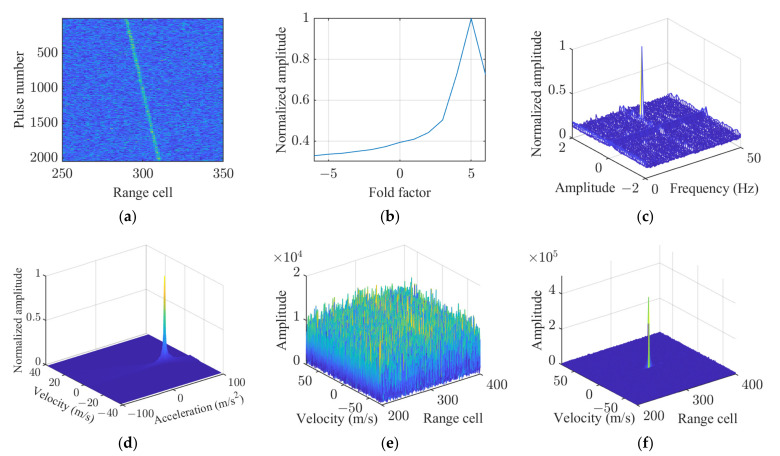
Simulation results of the KT-SRAF-LVD method. (**a**) Result after PC. (**b**) Fold factor search result. (**c**) Sinusoidal error parameters search result. (**d**) Acceleration estimation result. (**e**) Coherent integration result before correction. (**f**) Coherent integration result after correction.

After PC, the simulated signal exhibited an SNR of 6 dB, with significant RM observed in the echo signals, as illustrated in Figure 5a. The simulation results of the proposed KT-SRAF-LVD method are presented in Figure 5b–d. The results of coherent integration before and after correction are depicted in Figure 5e–f. It is evident that the SNR is significantly improved after RM and DFM corrections using the proposed method.

The simulation results demonstrate that the proposed method effectively compensates for both RM and DFM, enabling coherent integration.

### 5.2. Coherent Integration for Multiple Targets

The following simulation aims to verify the multi-objective processing capability of the method. The radar system parameters and sinusoidal error parameters are identical to those in Table 1 and Table 3, and the multiple equivalent motion parameters are summarized in Table 5.

**Figure 6:**
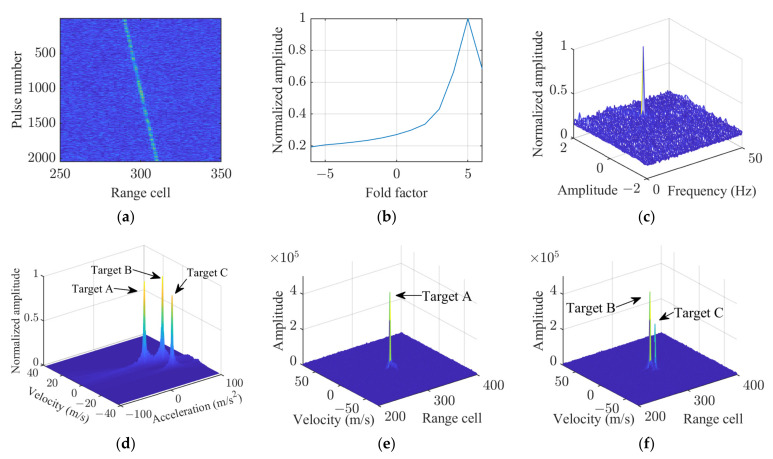
Simulation results of the KT-SRAF-LVD method for multiple targets. (**a**) Result after PC. (**b**) Fold factor search result. (**c**) Sinusoidal error parameter search result. (**d**) Acceleration estimation result. (**e**) Coherent integration result of target A. (**f**) Coherent integration result of targets B and C.

After PC, the simulated signal for each target exhibited an SNR of 6 dB, as shown in Figure 6a. Given the similarity in their motion parameters, the targets’ trajectories were closely clustered. Figure 6b presents the result of the fold factor search. The outcomes of the sinusoidal error parameter search are displayed in Figure 6c, where it is evident that cross-term interference had minimal impact on the results. Subsequently, after applying sinusoidal error compensation and the LVD method, the acceleration energy spectrum is shown in Figure 6d. The clear manifestation of three distinct energy peaks indicates the successful separation and identification of the multiple targets’ acceleration parameters. Coherent integration results of each target are shown in Figure 6e–f. Since targets B and C share the same range cell, fold factor, and acceleration, their coherent integration results can be represented in the same figure.

The simulation results demonstrate that the proposed method effectively suppresses the cross terms generated between multiple targets, showcasing its excellent performance in handling target interference.

### 5.3. Target Detection Performance

**Figure 7:**
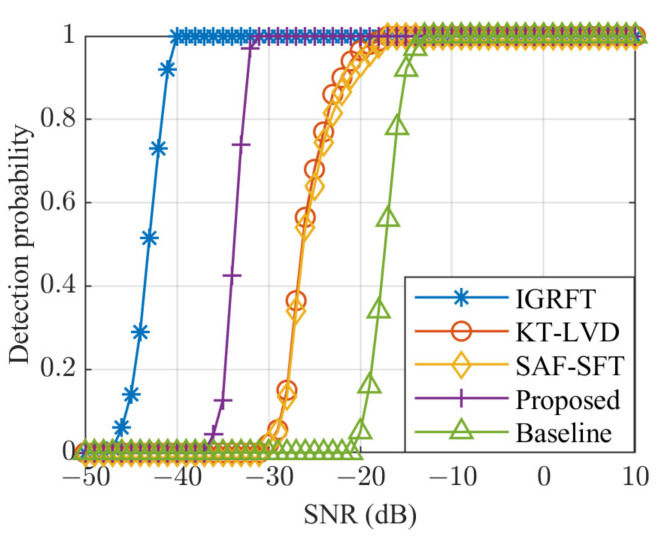
Detection probability of each method.

In this section, a CFAR detector is used to evaluate the detection performance of the IGRFT, KT-LVD, SAF-SFT, and the proposed method under Gaussian white noise conditions. To demonstrate the effectiveness of the proposed method, it is compared with the baseline approach, which is obtained without corrections for RM and DFM. The SNR ranges from −50 dB to 10 dB with an interval of 1 dB. For each SNR condition, 500 Monte Carlo simulations are performed. Under a false alarm probability of 10^−6^, the detection probabilities of each method as a function of SNR are shown in Figure 7.

As shown in Figure 7, the required SNR to achieve the desired detection probability ($P_{d}=1$) using the IGRFT method is approximately −40 dB, whereas the KT-LVD and SAF-SFT methods require around −17 dB. For the result without corrections, the required SNR is around −13 dB. The proposed KT-SRAF-LVD method achieves the same detection probability with a required SNR of approximately −31 dB. This corresponds to a detection performance loss of about 9 dB compared to the IGRFT method. Nevertheless, the proposed method demonstrates an improvement of 14 dB over both the KT-LVD and SAF-SFT methods and an improvement of 18 dB over the result without corrections.

The IGRFT method simultaneously searches for equivalent motion parameters and sinusoidal error parameters, achieving optimal detection performance. In contrast, the detection performance of the KT-LVD and SAF-SFT methods deteriorates without compensation for sinusoidal error and worsens further as the sinusoidal error increases.

## 6. Conclusions

In this paper, the challenge of excessive computational complexity in airborne coherent integration methods is addressed by improving the traditional KT-LVD method and proposing the KT-SRAF-LVD method. A radial rate echo model for high-speed maneuvering targets is established, and the compensation mechanisms for sinusoidal error and RM/DFM correction are theoretically derived. The method’s cross-term suppression capability and computational efficiency are analyzed in detail. Simulation experiments demonstrate the method’s effectiveness in both single-target and multi-target scenarios, demonstrating superior detection performance compared to existing methods such as KT-LVD and SAF-SFT, while maintaining a relatively low computational complexity. The KT-SRAF-LVD method successfully balances computational complexity and detection performance, demonstrating strong potential for real-time applications. While this study focuses on Gaussian white noise as the baseline scenario, future work will further explore the method’s adaptability to complex noise and clutter in real radar operating environments to comprehensively validate its practical application potential. Additionally, future work will explore hybrid frameworks combining KT-SRAF-LVD with AI techniques, such as using deep learning to refine the motion error compensation while preserving the interpretability of traditional signal processing.

## Figures and Tables

**Table 1:** Radar system parameters.

Parameter	Value
Carrier frequency	10 GHz
Pulse repetition frequency	10 KHz
Pulse width	20 µs
Bandwidth	10 MHz
Sampling rate	20 MHz
Pulse number	2048

**Table 2:** Equivalent motion parameters.

Parameter	Value
Initial range cell	300
Velocity	750 m/s
Acceleration	50 m/s^2^

**Table 3:** Sinusoidal error parameters.

Parameter	Value
Amplitude	0.8
Frequency	25 Hz

**Table 4:** Computational complexity of each method.

Method	Computational Complexity
Proposed	$O(M^{2}+M^{3}\log_{2}M+8M^{2}\log_{2}M+N_{n}M^{2}\log_{2}M)$
KT-LVD	$O(M^{2}+8M^{2}\log_{2}M+N_{n}M^{2}\log_{2}M)$
SAF-SFT	$O(M^{3}+M^{2}+8M^{2}\log_{2}M)$
IGRFT	$O(M^{6})$

**Table 5:** Equivalent motion parameters of multiple targets.

Parameter	Target A	Target B	Target C
Initial range cell	300	300	300
Velocity	750 m/s	750 m/s	740 m/s
Acceleration	20 m/s^2^	50 m/s^2^	50 m/s^2^

## Data Availability

Data are contained within the article.
